# Identification of *Wnt* Genes Expressed in Neural Progenitor Zones during Zebrafish Brain Development

**DOI:** 10.1371/journal.pone.0145810

**Published:** 2015-12-29

**Authors:** Robert N. Duncan, Samin Panahi, Tatjana Piotrowski, Richard I. Dorsky

**Affiliations:** 1 Department of Neurobiology and Anatomy, University of Utah, Salt Lake City, Utah, United States of America; 2 Stowers Institute for Medical Research, Kansas City, Missouri, United States of America; Universitat Pompeu Fabra, SPAIN

## Abstract

Wnt signaling regulates multiple aspects of vertebrate central nervous system (CNS) development, including neurogenesis. However, vertebrate genomes can contain up to 25 *Wnt* genes, the functions of which are poorly characterized partly due to redundancy in their expression. To identify candidate *Wnt* genes as candidate mediators of pathway activity in specific brain progenitor zones, we have performed a comprehensive expression analysis at three different stages during zebrafish development. Antisense RNA probes for 21 *Wnt* genes were generated from existing and newly synthesized cDNA clones and used for in situ hybridization on whole embryos and dissected brains. As in other species, we found that *Wnt* expression patterns in the embryonic zebrafish CNS are complex and often redundant. We observed that progenitor zones in the telencephalon, dorsal diencephalon, hypothalamus, midbrain, midbrain-hindbrain boundary, cerebellum and retina all express multiple *Wnt* genes. Our data identify 12 specific ligands that can now be tested using loss-of-function approaches.

## Introduction

Wnt/β-Catenin signaling is known to act in multiple ways to regulate vertebrate central nervous system (CNS) development, including as a mitogen [1], and in neural specification and differentiation [2–4]. The pathway also functions in post-embryonic neurogenesis, to promote the differentiation of neural progenitor cells in the dentate gyrus of the hippocampus [5], the rostral migratory stream [6], and the hypothalamus [4]. However with a few exceptions such as *wnt7a* in the dentate gyrus [7], it has been difficult to link functions in defined neural progenitor populations to specific Wnt ligands, possibly due to extensive redundancy within the Wnt family [8, 9]. In addition different Wnt ligands can activate multiple downstream pathways in the same tissue, such as in the zebrafish fin where *wnt10a* and *wnt5b* are both required for regeneration through ß-catenin-dependent and independent signaling, respectively [10]. It is therefore important to first precisely identify the specific Wnt ligands expressed in each neural progenitor population in order to test their function in neurogenesis.

Our laboratory is interested in the role of Wnt/ß-catenin signaling in hypothalamic neurogenesis, where we have previously shown a requirement for Lef1-mediated transcription in progenitor differentiation [4]. While we have identified one candidate ligand (*wnt8b*) in this region, knockout of this gene in mouse does not produce significant defects in brain development [11]. We thus decided to systematically examine the expression of the entire *Wnt* gene family in the developing zebrafish CNS in order to identify other candidates that may regulate hypothalamic neurogenesis. While many of these genes have been previously reported to have expression in specific brain regions (zfin.org), others have not been characterized and no single study has compared all the patterns at multiple stages.

For this work we examined the expression of 21 *Wnt* genes that either had known brain expression or were previously unexamined. While a comprehensive analysis of *Wnt* gene expression during early developmental stages has been previously performed [12], we carried out our experiments at 24, 48 and 72 hours post-fertilization (hpf), to cover both embryonic and post-embryonic CNS progenitor populations. At 48 and 72 hpf, we specifically focused on known progenitor zones including the telencephalic pallium/subpallium [13], the dorsal diencephalon (epithalimus and thalamus [14]), the ventral diencephalon (hypothalamus [4]), the midbrain [15], the midbrain/hindbrain boundary [16], the cerebellum [17], and the ciliary marginal zone (CMZ) of the retina [18]. Ultimately we were able to identify 12 genes with specific brain expression at all stages, most of which were localized to progenitor zones, and we found 3 genes (*wnt8b*, *wnt11r*, and *wnt16*) expressed in the hypothalamic posterior ventricular recess. Our results highlight the redundancy of Wnt ligand expression during zebrafish development, and lay the foundation for future functional analysis of Wnt signaling throughout the CNS.

## Materials and Methods

### Zebrafish embryo maintenance

This research was approved by the University of Utah IACUC Committee. All experiments were conducted on zebrafish embryos at 72 hours post-fertilization or younger, which are not considered to be vertebrate animals by IACUC guidelines. Zebrafish embryos were euthanized in tricaine. Fertilized wild-type (AB*) zebrafish embryos were and staged according to Kimmel et al. [19], and raised until 24, 48 and 72 hours post-fertilization (hpf). Embryos were fixed overnight at 4°C in 4% paraformaldehyde with 5% sucrose. Brains were manually dissected for in situ hybridization at 48 and 72 hpf.

### Cloning of zebrafish *Wnt* genes

The Ensembl *Danio rerio* genome database was used to identify genomic loci for all unpublished genes. Primers were designed to amplify ~500bp cDNA fragments for each gene (Table 1), and RT-PCR was performed on total RNA extracted from 24 hpf embryos using a Superscript II kit (Invitrogen). Amplicons were then subcloned into PCRII-TOPO (Invitrogen), and sequenced to verify gene identity as well as to confirm orientation for generation of antisense RNA probes.

**Table 1:** Sources of published in situ probe templates or primers used to amplify cDNA.

Gene	Reference or primers
*wnt1*	[20]
*wnt2*	[21]
*wnt2ba*	[21]
*wnt2bb*	[22]
*wnt3*	[23]
*wnt3a*	[20]
*wnt4a*	[24]
*wnt4b*	F: TGTATTTGATGTGTCGGCCA R: ACGCAGACACTTTGCCTTTT
*wnt5a*	F: ATGATGCTGCTGAAGCTGAAGT R: CTTACAGGTGTAAACCTCTTCTTTTTGT
*wnt5b*	F: GGAAGGATGGATGTGAGAATGAA R: CGTCTGCTACTTGCACACAAACT
*wnt7aa*	F: ATGAGCAGGAAAACGCGC R: TCACTTGCATGTGTACACTTCTGTC
*wnt7ba*	[25]
*wnt7bb*	[25]
*wnt8b*	[26]
*wnt9a*	F: GGAGAAGAAGCAGCGCAGAA R: CTTACAGGTGTAAACCTCTTCTTTTTGT
*wnt9b*	F: GGGATTTCAACACGGACAGATAG R: AAGCGCGTGAGACAATGCT
*wnt10a*	F: ATGAGCTCTCACGACATCAGTTG R: TGCTTGCTTATTTCATTTGCAGA
*wnt10b*	F: GTTCGACGCAATGGAGTTACC R: TGCTGCTCACTTGCACACATTA
*wnt11*	[27]
*wnt11r*	[28]
*wnt16*	Gift from Gilbert Weidinger

### In situ hybridization

Antisense riboprobes were synthesized and in situ hybridization was performed as previously described [29], with the addition of 5% dextran sulfate (Sigma-Aldrich D8906) to the hybridization buffer. To increase visualization of staining, some 24 hpf embryos were also incubated in 3%H_2_O_2_/0.5%KOH to remove pigmentation. Post-hybridization washes were carried out using a Biolane HTI in situ machine (Huller and Huttner AG). After staining, embryos were stored in MeOH and cleared in 70% glycerol for dissection and imaging. For sectioning, embryos were post-fixed in 4% paraformaldehyde for 1–2 hours at room temp, washed in PBS, and cryoprotected in 30% sucrose in PBS. Embryos were then embedded in OCT and sectioned at 20μm thickness on a Leica cryostat. Images were acquired using an Olympus BX51WI compound microscope and an Olympus Microfire camera. Digital images were cropped and aligned using Adobe Photoshop.

## Results

### Expression in the 24 hpf brain

**Fig 1:**
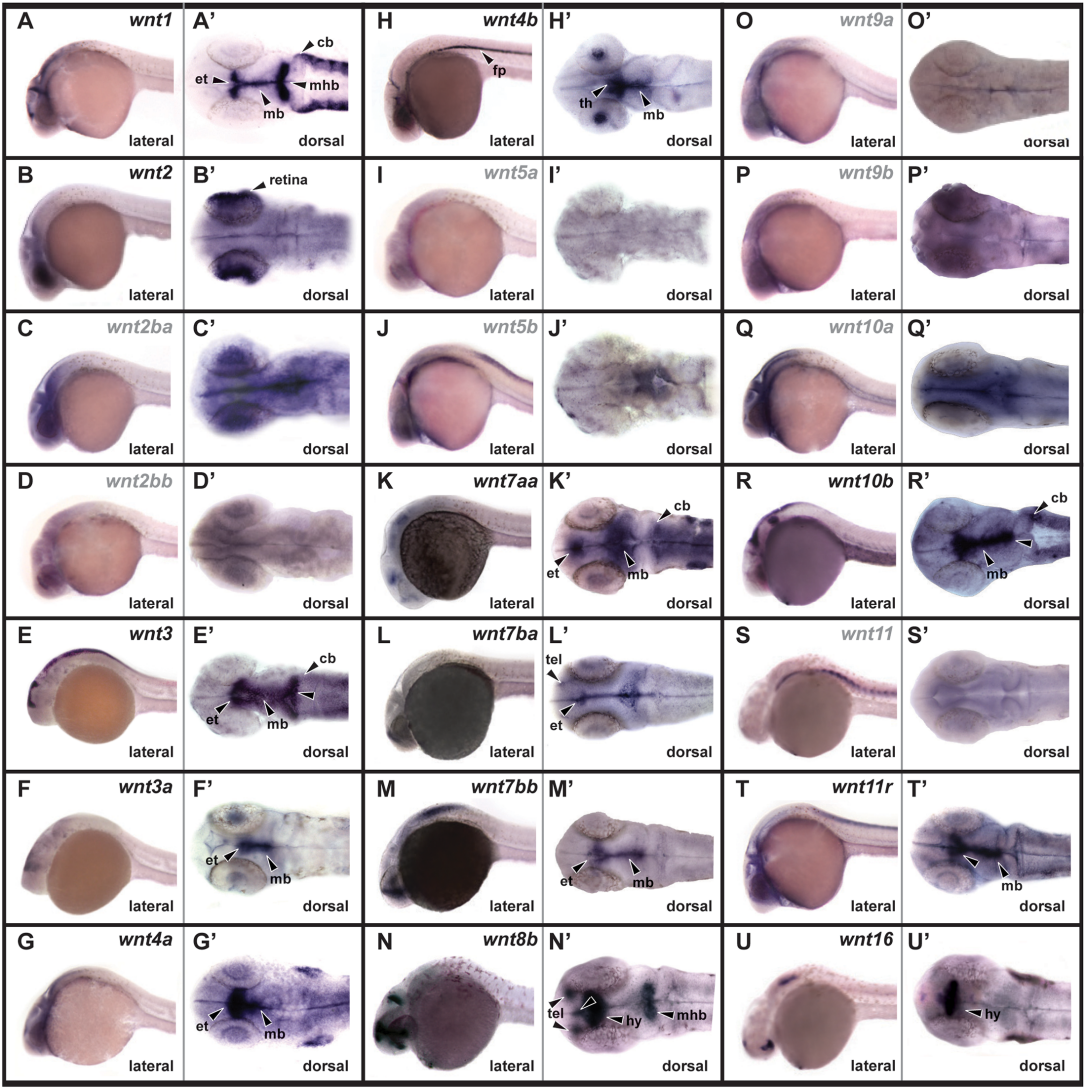
*Wnt* gene expression at 24 hpf. Lateral views of whole embryos are shown in left panels, and dorsal views of dissected brains are shown in right panels. Genes with nonspecific brain expression are indicated with grey text. tel, telencephalon; et, epithalamus; th, thalamus; hy, hypothalamus; mb, midbrain; mhb, midbrain-hindbrain boundary; cb, cerebellum; fp, floor plate.

We generated antisense probes for 21 zebrafish *Wnt* genes either from previously published DNA templates, or by RT-PCR amplification and subcloning (Table 1). The only known *Wnt* genes that we did not examine were *wnt7ab* and *wnt8a* due to their reported lack of CNS expression after somitogenesis [26, 30], and *wnt6a* and *wnt6b* due to their annotation after the initiation of this project. At 24 hpf much of the CNS is still rapidly proliferating and undergoing neurogenesis, and we observed expression of multiple *Wnt* genes expression throughout the brain (Fig 1 and Table 2). We found only two genes (*wnt7ba* and *wnt8b*) expressed in the telencephalon (Fig 1L' and 1N'). Eleven genes were expressed in the diencephalon, including seven in the epithalamus (Fig 1A', 1E', 1F', 1G', 1K', 1L', 1M' and 1N'), one (*wnt4b*) in the thalamus (Fig 1H'), and three (*wnt8b*, *wnt11r*, and *wnt16*) with expression in the hypothalamus (Fig 1N', 1T' and 1U'). Ten genes were expressed in the midbrain (Fig 1A', 1E', 1F', 1G', 1H', 1K', 1M', 1N', 1R' and 1T'), including four at the midbrain-hindbrain boundary (Fig 1A', 1E, 1N' and 1R'). Four genes were expressed in the rostral hindbrain (cerebellum) (Fig 1A', 1E', 1K' and 1R'). We also observed one gene (*wnt4b*) with expression in the floor plate of the hindbrain and spinal cord (Fig 1H). Nine other genes showed low-level ubiquitous or non-specific expression throughout the brain (Fig 1B–1D', 1I, 1J', 1O–1Q', 1S and 1S'), however we did observe specific expression of one gene (*wnt2*) in the retinal margin (Fig 1B').

**Table 2:** *Wnt* expression in the developing zebrafish brain.

	Telencephalon			Dorsal Diencephalon			Hypothalamus			Midbrain			Cerebellum			Retina (CMZ)	
	24 hpf	48 hpf	72 hpf	24 hpf	48 hpf	72 hpf	24 hpf	48 hpf	72 hpf	24 hpf	48 hpf	72 hpf	24 hpf	48 hpf	72 hpf	24 hpf	48 hpf
*wnt1*				**et**	**et**	**et**				**m**	**dm**	**dm**	**X**	**X**			
*wnt2*																**X**	**X**
*wnt2ba*																	
*wnt2bb*																	
*wnt3*				**et**	**et**	**et**				**m**	**dm**	**dm**	**X**	**X**	**X**		
*wnt3a*				**et**	**et th**	**et**				**X**	**d**	**d**					
*wnt4a*				**et**	**et**	**et**				**X**	**v**	**v**					**X**
*wnt4b*				**th**	**th**					**X**	**v**	**v**					**X**
*wnt5a*																	
*wnt5b*																	
*wnt7aa*		**p**	**p**	**et**	**et**	**et**				**X**	**d**	**d**	**X**	**X**	**X**		
*wnt7ba*	**X**	**p**	**p**	**et**	**et**	**et**					**d**	**d**					
*wnt7bb*		**p**		**et**	**et**	**et**				**X**	**d**			**X**			
*wnt8b*	**X**	**sp**	**sp**	**et**			**X**	**pr**	**pr**	**m**							
*wnt9a*																	
*wnt9b*																	
*wnt10a*																	
*wnt10b*					**et**	**et**				**m**	**dm**	**dm**	**X**	**X**	**X**		
*wnt11*																	
*wnt11r*							**X**	**pr**	**pr**	**X**							**X**
*wnt16*					**th**	**th**	**X**	**pr**	**pr**								

X, expression; p, pallium; sp, subpallium; et, epithalamus; th, thalamus; pr, posterior recess; d, dorsal; v, ventral; m, midbrain/hindbrain boundary.

### Expression in the 48 hpf brain

**Fig 2:**
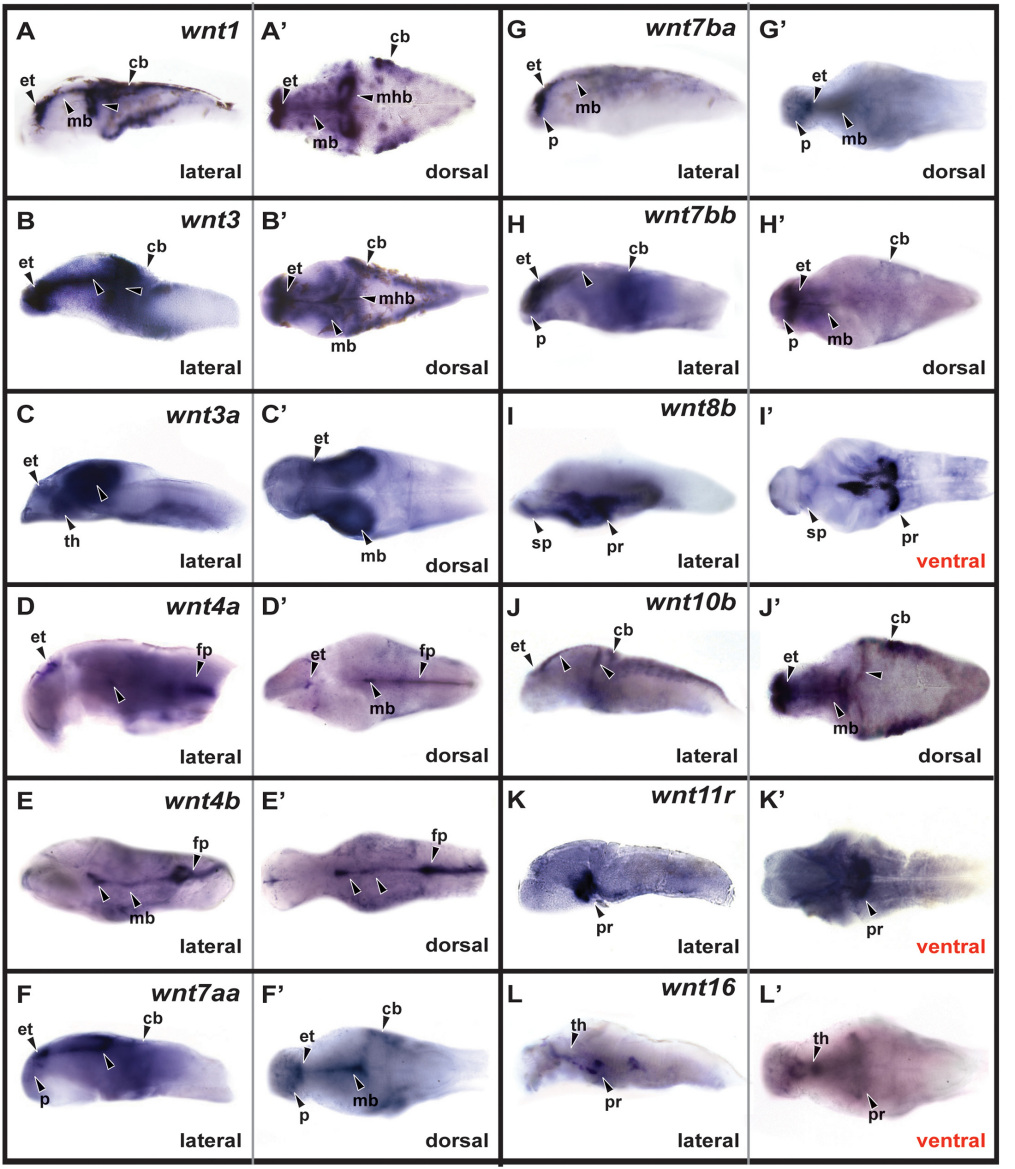
*Wnt* gene expression at 48 hpf. Lateral views of dissected brains are shown in left panels, and dorsal or ventral views of dissected brains are shown in right panels. p, pallium; sp, subpallium; et, epithalamus; th, thalamus; pr, posterior recess; mb, midbrain; mhb, midbrain-hindbrain boundary; cb, cerebellum; fp, floor plate.

**Fig 3:**
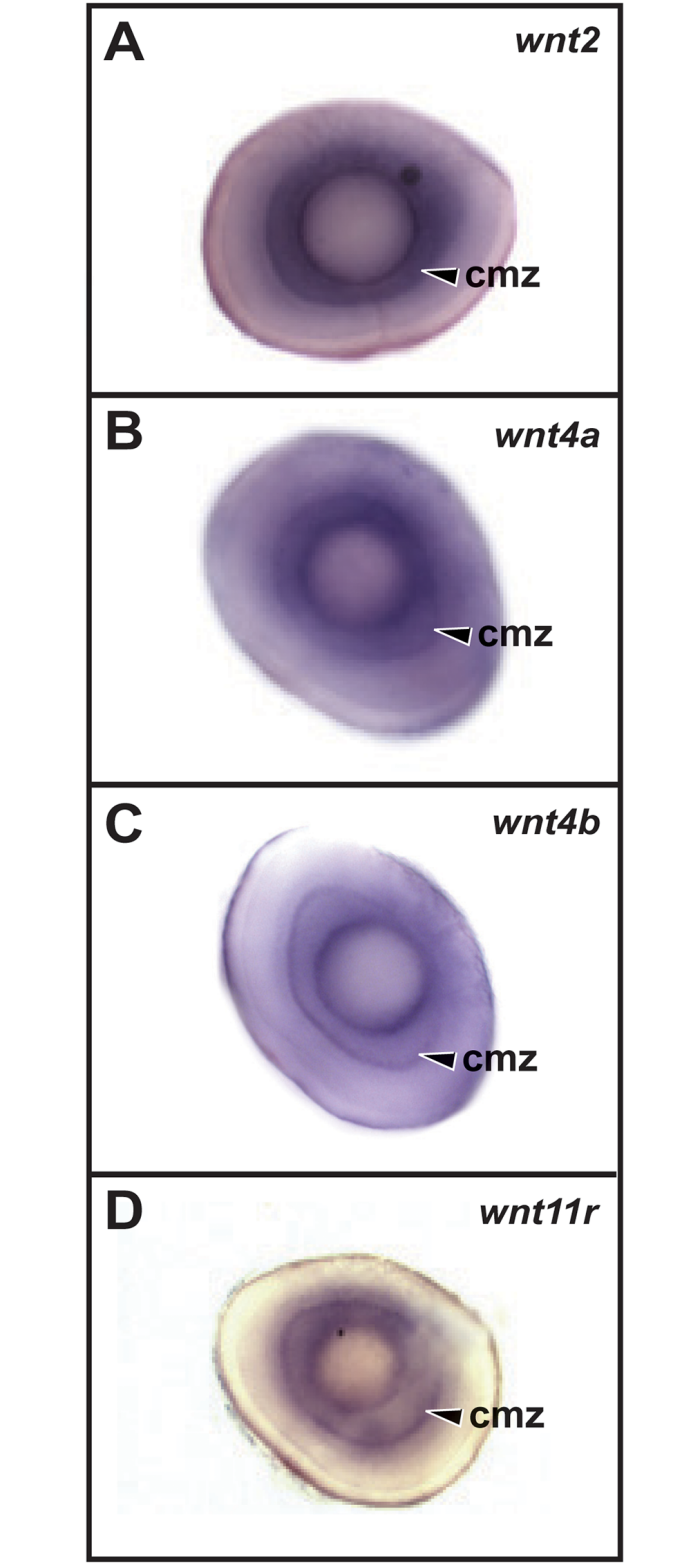
*Wnt* gene expression in the retina at 48 hpf. Dissected eyes are shown in all panels. cmz, ciliary marginal zone.

By 48 hpf the CNS has largely completed morphogenesis, and several proliferative neurogenic zones are retained near the ventricles [31]. At this stage and beyond we only analyzed expression patterns of the 12 genes that were specifically expressed at 24 hpf (Fig 2 and Table 2). In the telencephalon, expression of *wnt7aa*, *wnt7ba*, and *wnt7bb* was localized to the dorsal (pallial) region and *wnt8b* expression was localized to the ventral (subpallial) region (Fig 2F–2I'). In the dorsal diencephalon, eight genes were expressed in the epithalamus (Fig 2A–2D', 2F–2H', 2J and 2J'), and three genes (*wnt3a*, *wnt4b* and *wnt16*) were expressed in the thalamus (Fig 2B, 2B', 2E, 2E', 2L and 2L'). At 48 hpf the third ventricle of the hypothalamus has elaborated into bilateral recesses, and we observed specific expression of *wnt8b*, *wnt11r* and *wnt16* in the posterior recess as viewed from the ventral brain surface (Fig 2I, 2I' and 2K–2L'). Nine genes were expressed in the midbrain (Fig 2A–2H', 2J and 2J'), including three (*wnt1*, *wnt3*, and *wnt10b*) in the midbrain-hindbrain boundary (Fig 2A, 2B', 2J and 2J'), and two (*wnt4a*, *wnt4b*) in the ventral midline as well as the hindbrain floor plate (Fig 2D–2E'). The same four genes were expressed in the cerebellum as at 24hpf, with the addition of *wnt7bb* (Fig 2A–2B', 2F, 2F', 2H, 2H', 2J and 2J'). Finally, we observed expression of four genes in the ciliary marginal zone (CMZ) of the retina (Fig 3 and Table 2), which is the region containing neural progenitors [18].

### Expression in the 72 hpf brain

**Fig 4:**
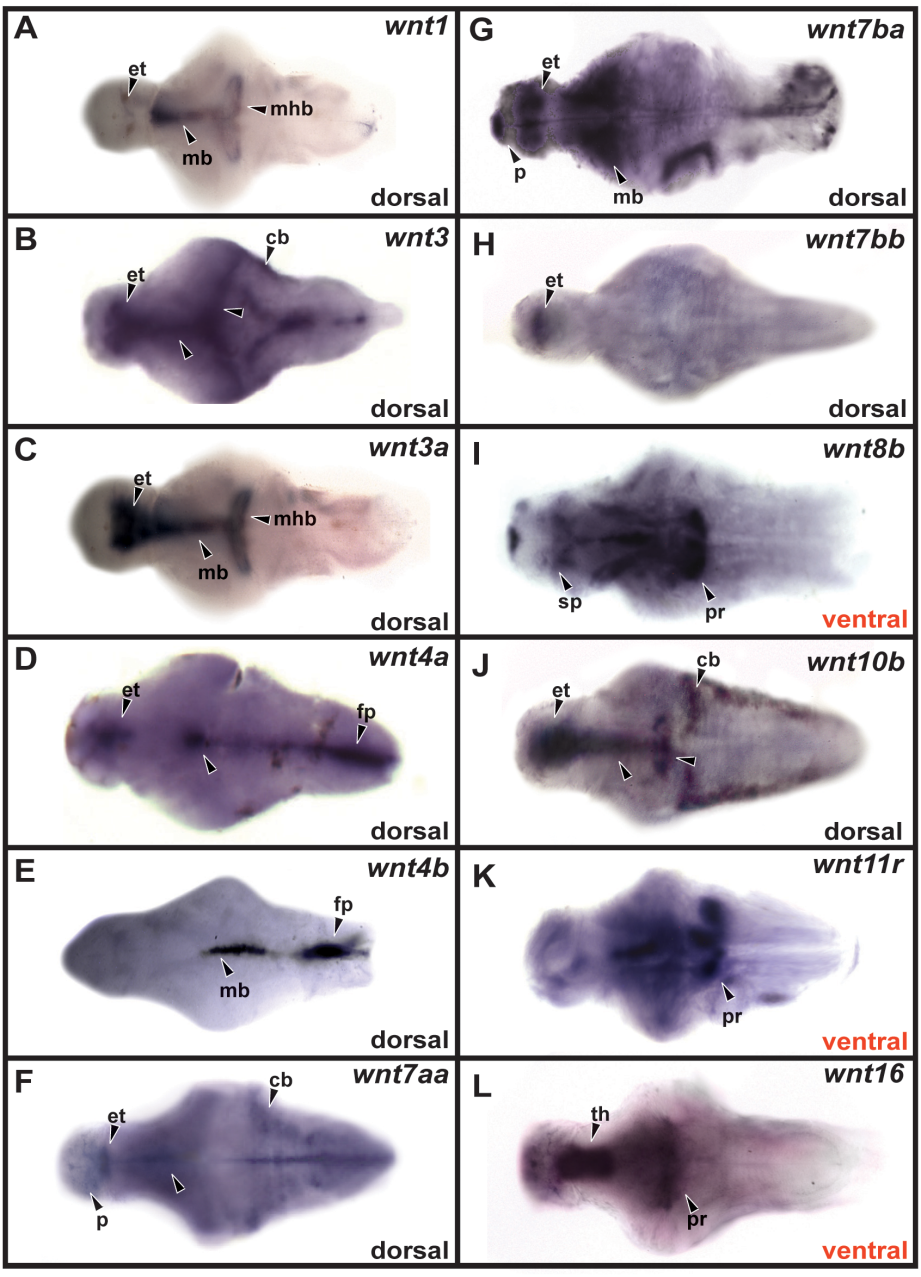
*Wnt* gene expression at 72 hpf. Dorsal or ventral views of dissected brains are shown in all panels. p, pallium; sp, subpallium; et, epithalamus; th, thalamus; pr, posterior recess; mb, midbrain; mhb, midbrain-hindbrain boundary; cb, cerebellum; fp, floor plate.

**Fig 5:**
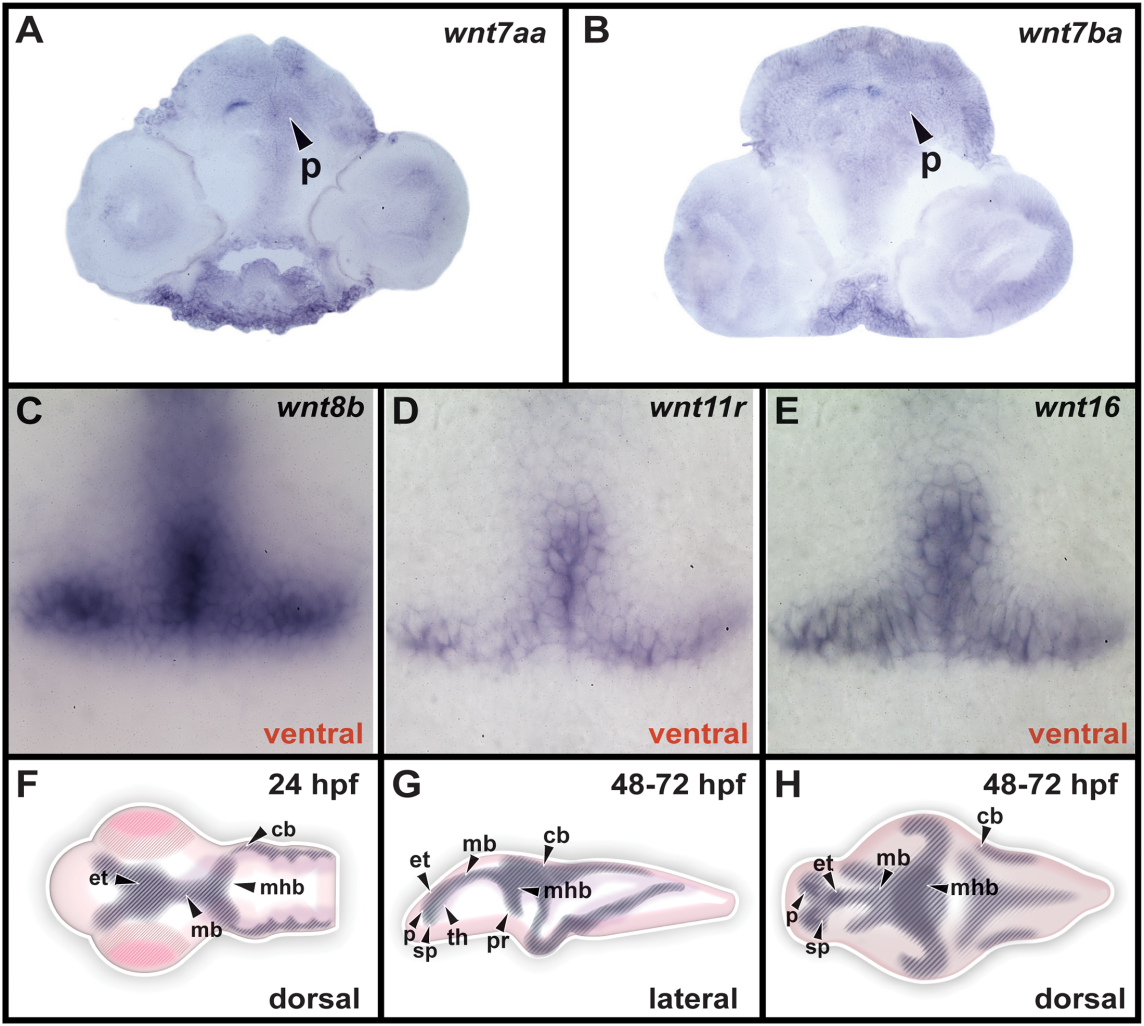
Specific expression of *Wnt* genes at 72 hpf and schematic diagram of brain anatomy. 20μm transverse cryosections at 72 hpf in panels A and B show expression in pallium (p). Ventral views of dissected brains in panels C-E show expression in the posterior hypothalamic recess (pr). Diagrams in panels F-H show regions of expression identified in Figs 1, 2 and 4.

Due to the larger brain size at 72 hpf, we were only able to characterize specific expression patterns using dorsal or ventral whole-mount views (Fig 4 and Table 2). We found that *wnt7aa* and *wnt7ba* were expressed in the pallium (Figs 4F, 4G, 5A and 5B), and *wnt8b* was still expressed in the subpallium (Fig 4I). The same eight genes were expressed in the epithalamus as at 48 hpf (Fig 4A–4D, 4F–4H and 4J), and *wnt16* continued to be expressed in the thalamus (Fig 4L). Similarly, *wnt8b*, *wnt11r*, and *wnt16* continued to be expressed in the hypothalamic posterior recess (Figs 4I, 4K, 4L and 5C–5E), and eight genes were expressed in the midbrain and midbrain-hindbrain boundary, (Fig 4A–4G and 4J). Expression of *wnt3*, *wnt7aa*, and *wnt10b* was observed in the cerebellum (Fig 4B, 4F and 4J) and expression of *wnt4a* and *wnt4b* was maintained in the floor plate (Fig 4D and 4E).

## Discussion

Our data clearly demonstrate the coincident expression of multiple *Wnt* genes in progenitor zones of the developing CNS (Fig 5F–5H and Table 2), and support previous observations of gene redundancy. Some of the overlapping gene expression that we observed could be explained by closely related orthologs arising from the teleost genome duplication [32], such as in the cases of *wnt4a/b* and *wnt7ba/bb*. However other related genes that are not teleost-specific duplicates, such as *wnt2/2b*, *wnt3/3a*, *wnt5a/b*, *wnt10a/b*, and *wnt11/11r*, have clearly different expression patterns indicating that their transcriptional regulation has likely diverged considerably. Investigations using double in situ hybridization and higher resolution analysis of specific CNS tissues can also be used to identify more subtle differences between grossly overlapping gene expression patterns. Expression of *Wnt* genes encoding ligands that signal through ß-catenin independent pathways also precludes simple predictions of redundant function. Importantly, both ß-catenin dependent and independent pathways play roles in stem cell maintenance and differentiation [33, 34], suggesting that post-embryonic neurogenesis may be regulated by multiple Wnt outputs in the same tissue.

The data presented here will be useful for the identification of candidate Wnt ligands that could mediate specific processes in CNS development, as demonstrated by the distinct subsets of *Wnt* genes expressed in consistent regional domains. For example, *wnt1*, *wnt3*, *wnt3a*, *wnt7aa*, and *wnt10b* are all localized to the roof plate of the diencephalon, midbrain and cerebellum. In contrast, *wnt4a* and *wnt4b* show consistent expression in floor plate structures, and *wnt8b*, *wnt11r*, and *wnt16* are expressed in the hypothalamus. In addition, our analysis offers a starting point for further studies investigating the functions of Wnt ligands in developmental and post-embryonic neurogenesis. The identification of three ligands expressed in the hypothalamic posterior recess provides specific targets for manipulating Wnt pathway activity in a defined model of neural progenitor maintenance and differentiation, especially as mutant alleles are available for all three genes [35]. With the recent development of effective gene targeting methods in zebrafish [36], it will be possible to produce animals carrying null alleles for all the potential *Wnt* genes expressed in or near a given CNS tissue or cell population.
